# Giant Cervico-Thoracic Cystic Hygroma in a Preterm: A Case Report

**DOI:** 10.21699/jns.v6i3.593

**Published:** 2017-08-10

**Authors:** Rahul Mansing Kadam, A Narendra Kumar, VSV Prasad, Sudha Boda

**Affiliations:** Lotus Hospitals for Women and Children, Lakdikapul, Hyderabad, Telangana, India

**Keywords:** Cystic hygroma, Surgical excision, Hydrops, Turner syndrome, Sclerotherapy.

## Abstract

Cystic hygroma is a benign, painless loculated lymphatic proliferation, which occur due to a combination of sequestration from developing lymphatic system, abnormal budding of the lymphatic system or lack of development of the normal connections between venous and lymphatic drainage. We report a case of giant cervico-thoracic cystic hygroma in a preterm neonate with management options and a brief review of literature.

## CASE REPORT

A preterm (female, 33 weeks, 3.5 kg) 1st of the twins, was born by emergency LSCS. The other twin was male with weight of 1.4 kg. Antenatal scan was suggestive of cystic hygroma in the neck region. At birth, she was apneic and required bag and tube ventilation. She developed respiratory distress soon after. She had giant cystic swelling over neck on right side extending to axilla and trunk on right side (Fig.1A) with skin ulceration. There was gross tilting of the neck to the right side. There were no other obvious congenital abnormalities. Decompression of the swelling was performed by needle aspiration as an emergency procedure and 1.5 liters of hemorrhagic fluid was drained. Post-decompression her weight was 1.6 kg (Fig.1B). She was extubated and weaned to nasal CPAP after initial stabilization. Skiagram of neck and chest was suggestive of cervico-axillary soft tissue mass with features suggestive of RDS. No rib cage deformities were noted. USG neck and chest revealed presence of large well defined anechoic lesion with septations within; along the nape of neck, right axillary, right lateral thoracic wall along soft tissue planes outside bony thoracic cage- features suggestive of cystic hygroma. Echocardiography revealed tiny closing PDA.

After optimization, surgical excision of the cystic hygroma was performed on day 2 of life under general anesthesia; a long incision was given extending from right post-auricular area to mid-supraclavicular area. Skin and subcutaneous tissue were separated from the cyst wall. The cyst was isolated from the surrounding structures anteriorly and laterally. With traction on the cyst wall the medial exploration was performed to separate the cyst wall from vital vascular and neural structures. The cyst was extending into the carotid sheath and was extended up to pharynx and tracheal wall but not invaded the wall. After completely mobilizing the cervical component, a separate horizontal incision was given for excising the right axillary component. The axillary component was found to be extending on to the right mammary area anteriorly to the scapular area posteriorly. After adequate mobilization, both the cervical and axillary components were removed in toto. The cervical and axillary incisions were closed separately after keeping separate suction drain tubes (Fig.1C). Post-operatively, she required mechanical ventilation for three days after which she could be weaned to room air.

Orogastric tube feeds were initiated on 2nd post-operative day and advanced to oral feeds on day 21 of life. Her chromosomal analysis was normal. On follow-up, her surgical wound has healed well (Fig.1D). She is doing well with normal growth and development.

**Figure:**
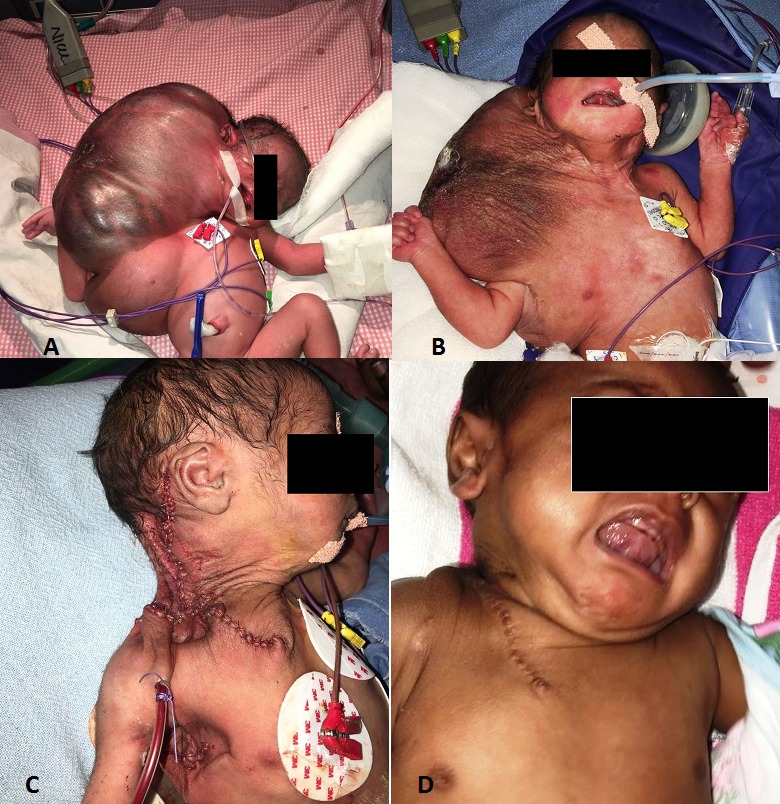
Figure 1: A. Giant cervico-axillary cystic hygroma, B. After aspiration with needle, C. Post excision, D. On follow-up showing good healing.

## DISCUSSION

Antenatally detected cystic hygromas have been associated with poorer prognosis, although up to 42% of these may resolve spontaneously by birth [1]. About 30 to 70% of fetuses with cystic hygroma have chromosomal aberrations. Turner syndrome being most common (60% of cases) [2] followed by Down syndrome and Trisomy 18 [3]. Fetal karyotyping is recommended and serial ultrasounds should be performed every 3-4 weekly to see the progress. The most common sites include the posterior neck (75%) and axilla (20%) followed by mediastinum, groin, bones, retroperitoneum, abdominal viscera, and scrotum. Most common associated structural malformations are cardiac; fetal echocardiography is recommended. Complications of cystic hygromas are airway obstruction, dysphagia, bleeding into the cyst, infections and abscess formation.

If the cystic hygroma is giant lesion as in our case, the mode of delivery should be a planned caesarean section at term gestation to avoid rupture of the lesion during delivery. In our case, it was emergency LSCS in view of preterm onset of labor pains and twin gestation. Surgical excision remains the mainstay of treatment for cystic hygroma. It is preferable to have surgical access through two different incisions; cervical vertical incision and axillary horizontal incision for excision of the lesion in toto, as in our case. Alternatives to surgery include injecting sclerosing agents into the mass and laser therapy [4]. Intralesional bleomycin injection can be used in patients with large cystic masses and extensive invasion to reduce the risk of injury to vital organs [5]. Another sclerosing agent is OK-432, which causes less fibrosis of the subcutaneous tissue and the overlying skin, and is cosmetically better. Doxycycline is a safe and effective sclerosant agent for treating cystic hygromas in children, with a low complication rate [6]. However, sclerotherapy is not free of complications and has many adverse effects which include swelling at the site of the lesion which further compromises airway, scarring, pulmonary fibrosis, hemorrhage, and infection [7]. Sclerosing agents are known of causing scarring and contraction of the surrounding tissues, rendering subsequent surgery more difficult. Other complications include slow regression of lesions leading to longer duration of treatment, risk of recurrence and the long-term toxicities of the treatment are not known [8].

As in our case, in twin gestation because of the giant nature of the lesion and space constraint within the uterine cavity, the affected infant’s growth may be compromised and there is need of taking care of nutritional requirement of the affected infant. In view of giant nature of the lesion, preterm infant with a respiratory compromise and potential risk of toxicity we opted for surgical excision. Furthermore, safety profile of the sclerosants in preterm infants is not well known.

## Footnotes

**Source of Support:** None

**Conflict of Interest:** None
